# Maternal perceptions and concerns about children’s weight status and diet quality: a study among Black immigrant families

**DOI:** 10.1017/S1368980021004729

**Published:** 2022-08

**Authors:** Cris-Carelle Kengneson, Rosanne Blanchet, Dia Sanou, Malek Batal, Karen P Phillips, Isabelle Giroux

**Affiliations:** 1 Interdisciplinary School of Health Sciences, Faculty of Health Sciences, University of Ottawa, Ottawa, ON, Canada; 2 Department of Social and Preventive Medicine, School of Public Health, Université de Montréal, Québec, Canada; 3 FAO Sub-Regional Office for Eastern Africa, Food and Agricultural Organizations of the United Nations, Addis Ababa, Ethiopia; 4 Nutrition Department, Faculty of Medicine, Université de Montréal, Centre de recherche en santé publique (CReSP), Québec, Canada; 5 School of Nutrition Sciences, Faculty of Health Sciences, University of Ottawa, Ottawa, ON K1N 6N5, Canada; 6 Institut du Savoir Montfort, Ottawa, ON, Canada

**Keywords:** Perceptions, Concerns, Weight status, Diet quality, Black immigrants

## Abstract

**Objective:**

To identify factors influencing Black immigrant mothers’ perceptions and concerns about child weight and to compare children’s diet quality according to these perceptions and concerns.

**Design:**

Mothers’ perceptions and concerns about child weight were assessed with sex-specific figure rating scales and the Child Feeding Questionnaire, respectively. Participants’ weights and heights were measured and characterised using WHO references. Children’s dietary intakes were estimated using a 24-h dietary recall. Children’s diet quality was evaluated using the relative proportion of their energy intake provided by ultra-processed products, which were identified with the NOVA classification. *χ*^2^ tests, multivariate logistic regressions and *t* tests were performed.

**Setting:**

Ottawa, Ontario, Canada.

**Participants:**

Black immigrant mothers of Sub-Saharan African and Caribbean origin (*n* 186) and their 6–12-year-old children.

**Results:**

Among mothers, 32·4 % perceived their child as having overweight while 48·4 % expressed concerns about child weight. Girls and children with overweight or obesity were significantly more likely to be perceived as having overweight by their mothers than boys and normal-weight children, respectively. Mothers of children living with obesity, but not overweight, were significantly more likely to be concerned about their child’s weight than mothers of normal-weight children. Children’s diet quality did not differ according to mothers’ perceptions and concerns.

**Conclusions:**

Children’s gender and weight status were major determinants of perceptions and concerns about child weight among Black immigrant mothers. Including knowledge about mothers’ perceptions and concerns about child weight will help nutrition professionals develop interventions tailored to specific family needs within the context of their cultural backgrounds.

Childhood obesity is a major public health issue in Canada given its widespread prevalence and negative impact on health^(1)^. Genetic, environmental and socio-ecological factors contribute to the development of childhood obesity^(2)^ with parents establishing the food environment at home, thereby influencing children’s food choices, eating habits and energy intake^(3)^. Consequently, it is recommended that parents be involved in interventions to prevent and/or mitigate childhood obesity^(4)^. Engagement of parents requires an understanding of parental perceptions and practices regarding child weight and feeding as they may have an impact on the effectiveness of these interventions^(2)^.

Parental perceptions of their children’s weight influence children’s weight over time. Indeed, longitudinal studies suggest that children whose parents perceived them as having overweight or obesity were more likely to gain and maintain excess weight over time^(5–7)^. One study showed that children living with overweight or obesity who were perceived as having overweight or obesity by their parents were more likely to have a higher increase in BMI from age 5 to 9 compared to children with overweight or obesity who were not perceived as such^(5)^. An Australian longitudinal study reported that parental perceptions of overweight among children predicted more weight gain across 8 years of follow-up compared with parental perceptions of normal weight among children^(6)^. Similarly, Robinson and Sutin^(7)^ examined two different cohorts (age: 4–5 to 14–15 and 9–13 years old) and found that parental perceptions of overweight were associated with greater weight gain during follow-up. Interestingly, results from these three studies were significant whether parental perception was accurate or not, meaning that normal-weight children and children with overweight or obesity who were perceived as having overweight or obesity by their parents gained more weight than children who were not perceived as having overweight or obesity^(5–7)^. Authors suggested that parental perception of overweight and obesity could increase the risk of weight gain because of the stigma associated with this perception^(6–8)^, which may result in the adoption of counterproductive strategies to reduce children’s weight^(6,7)^, such as the use of restrictive feeding practices. These counterproductive feeding practices are associated with excessive weight gain in the long run^(9,10)^. Children perceived as having overweight or obesity are also more likely to perceive themselves as having overweight or obesity and to unsuccessfully attempt to lose weight^(7,11)^. Consequently, these studies highlight the need to shift the field from understanding determinants of parental misperception (or underestimation) towards understanding determinants of parental perception of children’s weight per se (i.e. whether it is accurate or not)^(5–7)^. Authors of a recent systematic review reached a similar conclusion^(12)^. Yet, to our knowledge, no study has examined factors associated with parental perceptions of children’s weight status per se.

Parental practices regarding child weight and feeding also seem to be associated with parental concerns about child weight^(3,13,14)^. Parental concerns about child weight were associated with parents’ use of strategies to promote healthy child weight, such as increasing physical activity or limiting screen time^(13)^. However, the literature also suggests that mothers who were concerned about their children’s weight and risk of gaining excess weight were more likely to use restrictive and pressure to eat feeding practices^(3,14)^. These feeding practices were previously determined not only to have a negative impact on children’s diet quality but were also associated with excessive weight gain over time^(9,10,15)^. Although parents whose children have overweight or obesity are more likely to be concerned about their children’s weight, children’s age^(16,17)^ and gender^(13,18,19)^, as well as parental weight status^(19–21)^, region of origin^(22)^ and education level^(23)^ also contribute to these concerns. In contrast, parental age, socio-economic status and marital status were found not to be associated with parental concerns about child weight^(3,18,21)^.

Children’s diet quality can be assessed by quantifying food intake and comparing it with dietary intake recommendations^(24)^. However, with the increasing availability and consumption of ultra-processed products (UPP), such as junk food, researchers have started to focus on food processing and its impact on health^(25–27)^. UPP have poor nutritional values with UPP intake identified as an indicator of poorer diet quality^(27)^.

Published studies examining parental perceptions and concerns related to children’s weight status have been conducted among various populations, but mainly among White families^(12,22)^. Studies have suggested that Black parents were less likely to perceive their child as having overweight or obesity and less likely to be concerned about child weight compared with White or Latino parents^(28–30)^. However, these studies were mostly conducted in the USA with relatively small samples of African Americans and/or Blacks. Similarly, studies on UPP consumption have been mainly conducted among Latino and White populations^(31,32)^. In Canada, Black people represent 3·5 % of the population. In Canada, most Black people are immigrants (56·4 %) or children of immigrants (35·0 %), primarily from Sub-Saharan Africa and the Caribbean^(33)^. Thus, the experience of most Black people in Canada is shaped by their intersecting identities as racialised minorities and by their diverse immigration experience (or that of their parents)^(34)^. Upon arrival to Canada, Black immigrants face several discrimination-related challenges particularly regarding foreign credential recognition, employment and housing which often lead to a lower socio-economic status than the main population, and consequently, a deterioration of their health^(35,36)^. For example, Black immigrants generally exhibit better health than Canadian-born upon arrival; however, their risk of developing chronic diseases such as obesity increases over time and surpasses that of their Canadian counterparts^(37,38)^. Determinants of Black immigrant people’s health, including factors associated with parental perceptions and concerns about their children’s weight status, are under-researched in Canada and worldwide^(34)^. Further, examinations of feeding practices, perceptions and concerns within Black families have generally not considered the heterogeneity within Black communities, including factors such as immigration status. The heterogeneity of both the African and Caribbean diaspora precludes an in-depth evaluation of the roles of culture and heritage on feeding practices, perceptions and concerns. As such, findings from other studies are limited and not generalisable to the Canadian context because of the interplay of ethnicity, migration and socio-economic factors. Therefore, this research was designed to explore the relationships between maternal immigration experiences and children’s weight status in Black Sub-Saharan-African and Caribbean mothers living in Canada. More specifically, the purpose of this study was to: (1) identify factors influencing Black immigrant mothers’ perceptions and concerns about child weight and (2) compare children’s energy intake and diet quality according to mothers’ perceptions and concerns about child weight.

## Methods

### Participants

Mothers were recruited in Ottawa, Canada, between January 2014 and April 2015 through word of mouth, community events and with the help of ethnocultural associations and community organisations^(39)^. Mothers, not fathers, were recruited in this study due to their preponderant implication in children’s diet in this population^(40,41)^ and the high prevalence of lone mothers in these groups due in part to immigration and consequent family separation^(42)^. Mothers were included if they self-identified as Black; were born in Sub-Saharan Africa or the Caribbean; could have a conversation in English or French; had at least one child aged 6–12 years and resided in Ottawa. When more than one school-age child from the same household was eligible, one child was randomly selected. Three mothers born in Canada were included as they had African or Caribbean parents and spent their childhood in Sub-Saharan Africa or the Caribbean before coming back to Canada as adults. The sample included 188 mother–child dyads.

### Procedure

Surveys were administered in-person by registered dietitians to mother–child dyads with the help of a trained research assistant when possible. Surveys were conducted in English or French, according to mothers’ language preferences. Each mother received a $25 CAD grocery gift certificate as compensation for the time and the cost involved in their participation.

### Positionality

Authors from this study position themselves as academics and community researchers with great research interests and experience working with populations at high risk of health inequities, including minority and racialised communities. We share a commitment to nutrition and health equity and strongly believe everyone in Canada should have equal access to conditions conducive to good nutrition and health. Our interdisciplinary team is composed of Sub-Saharan African Black immigrants, Canadians, Lebanese-Canadian and first-generation Caribbean-Canadian with different experiences and expertises, which could have influenced our perspective and approach to this study.

### Measures

#### Demographic and socio-economic data^(43)^

Mother (age, region of origin, marital status, education level, employment status, immigration category, length of time spent in Canada), child’s (gender, age) and household (number of children in the household, income, receipt of social or government assistance, food insecurity status) characteristics were collected using surveys. The Household Food Security Survey Module was used to assess household food insecurity status based on its eighteen items^(43)^. Households with no affirmative answers were categorised as food secure while households with one or more affirmative answers were categorised as food insecure^(44)^. Mothers’ arrival date in Canada and the interview date were used to calculate each mothers’ length of time spent in Canada and to classify them as recent immigrants (≤ 5 years) or settled immigrants (≥ 5 years).

#### Children and maternal anthropometric data

Mothers’ and children’s anthropometric data were measured according to the WHO guidelines^(45)^. Weight was measured with a calibrated digital scale (LifeSource ProFit UC-321, A&D Medical) to the nearest 0·1 kg. Height was measured with a portable stadiometer (Charder HM200P Portstad, Charder Electronic Co.) to the nearest millimetre. Measurements were done twice and averaged to increase measurement reliability. Participants’ BMI was calculated (kg/m^2^). Mothers’ weight status was obtained using the WHO references^(45)^. WHO Growth Charts for Canada were used to assess BMI-for-age-and-sex *z*-scores then children’s weight status^(46)^. Underweight categories from the study were excluded for mothers and children because only one mother and one child were classified as such, which left us with a final sample of 186 dyads included in this study. Mothers who were breast-feeding or pregnant (*n* 9) were excluded from anthropometric analyses only. Anthropometric data were missing for ten mothers and two children. Therefore, anthropometric data of 167 mothers and 184 children were available for analysis.

#### Body shape perceptions

Age-appropriate and sex-specific figure rating scales adapted by Stevens *et al*.^(47)^ were used to assess mothers’ perception of their children’s body shape. The eight silhouettes were grouped into four categories: underweight (silhouettes #1 and #2), normal weight (silhouettes #3 and #4), overweight (silhouettes #5 and #6) and obesity (silhouettes #7 and #8). Maternal perceptions of obesity were merged into perceptions of overweight as only three children were perceived as having obesity by their mothers. We also combined underweight and normal weight categories to increase statistical power^(48)^. Mothers’ misperception of children’s weight status was determined by comparing perceived and measured children’s weight status (see online Supplementary Material).

#### The Child Feeding Questionnaire

The Child Feeding Questionnaire was used to assess maternal concerns about her child’s risks of becoming overweight^(49)^. Translation and back translation were used to obtain a French version of the Child Feeding Questionnaire^(50)^. The three Child Feeding Questionnaire’s questions related to parental concerns about child weight (i.e. *How concerned are you about your child eating too much when you are not around? How concerned are you that your child will have to diet to maintain a desirable weight? How concerned are you about your child’s weight in the future*?) were answered on five-point Likert scales ranging from unconcerned to very concerned. The score of each question was added, and an average score was calculated^(49)^. To facilitate interpretation, scores were dichotomised as unconcerned (scores < 2) and concerned (scores ≥ 2) based on the distribution to have two groups of similar size.

#### Children’s diet quality

Children’s food intake was assessed with a 24-h dietary recall using the Health Canada standardised protocol^(51)^. Questions were directed at children, and mothers could intervene to provide additional information about food preparation and ingredients. The 24-h dietary recalls were analysed with ESHA Food Processor SQL version 11.0.137 (ESHA Research). Nutrient contents, including the total energy intake in kilocalories (kcal) of the foods and beverages consumed, were obtained from the Canadian Nutrient File^(52)^ and other sources when needed (e.g. US Department of Agriculture database). The NOVA Food Classification system was used to assess children’s diet quality in relation to foods and beverages’ level of processing^(53)^. In this system, foods are classified into four groups according to their level of processing: (1) unprocessed or minimally processed foods, which are fresh or whole foods such as fruits, vegetables, meats and eggs with no industrial processing, (2) processed culinary ingredients such as salt, sugar and oils often extracted and refined from unprocessed or minimally processed foods or from nature, (3) processed foods which are foods minimally processed to increase their durability or to make them more palatable (e.g. simple cheeses, cured meats, preserved fruits and vegetables) and (4) UPP, which are industrial formulations derived from foods and containing ingredients and substances such as colourings, emulsifiers or artificial flavours that are not found in a traditional kitchen (e.g. soft drinks, industrial bread, confectioneries, etc.)^(53)^. Dietary supplements were excluded from the classification. The relative consumption of the total daily energy intake (in percentage) provided by unprocessed or minimally processed foods and by UPP groups was estimated^(54)^.

### Data analysis

SPSS IBM for Windows version 25 was used for statistical analyses^(55)^. Means, standard deviations and frequencies (%) were used to describe data. Pearson *χ*^2^ tests coupled with Bonferroni *post hoc* tests were performed to examine factors associated with maternal perceptions and concerns about child weight. Factors examined were children’s age, gender and weight status, mothers’ age, marital status, weight status, employment status, education level, immigration status, length of time spent in Canada, region of origin and household income, receipt of social or government assistance, number of children in the household and food insecurity status. Variables with a *P* value < 0·25 in Pearson *χ*^2^ tests were included in multivariable regression models^(56)^ to identify predictors of maternal perceptions of children’s weight status (1 = child perceived as having overweight, 0 = child perceived as normal weight) and concerns about child weight (1 = concerned, 0 = unconcerned). Children’s weight status was subsequently included in models due to its known effect on parental perceptions and concerns about child weight. OR, 95 % CI and *P* value were calculated for every model. To examine the robustness of results related to concerns about child weight, we conducted multivariate regression models using continuous scores to examine whether the same patterns of results would be identified, and this was broadly the case (data not shown). *t* Tests were conducted to compare children’s daily energy intake and diet quality according to mothers’ perceptions of children’s weight status and concerns about child weight^(57)^. Factors influencing mothers’ misperceptions of children’s weight status and comparison of children’s daily energy intake and diet quality according to mothers’ misperceptions of children’s weight status were also studied, and results are presented in online Supplementary Material. All assumptions for *χ*^2^ tests, regression models and *t* tests were checked and respected. Significance level was set at *P* < 0·05 for all analyses.

## Results

### Sample characteristics

The sample comprised mostly settled immigrant participants (66·5 %) born in Sub-Saharan Africa (66·7 %) and the Caribbean (33·3 %) (Table 1). An important proportion of the sample experienced household food insecurity (44·0 %) and were refugees or asylum seekers (38·4 %). Children were on average 8·8 (sd 1·98) years old and over 54 % of mothers were under 40 years old. The sample comprised a similar proportion of girls (50·5 %) and boys (49·5 %). The combined prevalence of childhood overweight and obesity was 48·9 %. One-third of mothers (32·3 %) perceived their children as having overweight. Children’s weight status was misperceived by 51·6 % of mothers, with 47·8 % underestimating their children’s weight status (see online supplementary material, Supplemental Table 1).

**Table 1:** Association between participants’ characteristics and mothers’ perception of children’s weight status

			Participants’ characteristics										
Perception of children’s weight status (*n* 186)	All (*n* 186)		UW/NW *n* 126 (67·7)		OW *n* 60 (32·3)			Model 1 Perception of children’s weight status¶ *n* 161			Model 2 Perception of children’s weight status¶ *n* 160		
	*n*	Percentage of participants	*n*	Percentage of participants	*n*	Percentage of participants	*P*	OR**	95 % CI	*P*	OR**	95 % CI	*P*
Child’s gender													
Girls	94	50·5	55	43·7^a^	39	65·0^a^	0·006	Reference			Reference		0·041
Boys	92	49·5	71	56·3^b^	21	35·0^b^		0·32	0·15, 0·71	0·005	0·37	0·14, 0·96	
Number of children in the household													
Two or less	88	47·3	55	43·7	33	55·0	0·147	Reference					
Three or more	98	52·7	71	56·3	27	45·0		0·52	0·25, 1·11	0·090			
Child’s age (years)													
Less than 9	83	44·6	59	46·8	24	40·0	0·381						
9 and older	103	55·4	67	53·2	36	60·0							
Mothers’ age (years)													
Less than 40	101	54·3	69	54·8	32	53·3	0·855						
40 and older	85	45·7	57	45·2	28	46·7							
Children’s weight status (*n* 184)													
NW	94	51·1	88	70·4^a^	6	10·2^a^	< 0·001				Reference		
OW	44	23·9	28	22·4^b^	16	27·1^b^					11·78	3·36, 41·33	< 0·001
OB	46	25·0	9	7·2^c^	37	62·7^c^					46·22	13·104, 163·00	< 0·001
Mothers’ weight status (*n* 167)													
NW	28	16·8	23	20·4^a^	5	9·3^a^	0·012	Reference			Reference		
OW	61	36·5	46	40·7^a,b^	15	27·7^a,b^		1·84	0·50, 6·70	0·357	1·26	1·27, 5·97	0·770
OB	78	46·7	44	38·9^b^	34	63·0^b^		6·41	1·84, 22·37	0·004	2·50	0·55, 11·46	0·237
Mothers’ region of origin													
Sub-Saharan Africa	124	66·7	84	66·7	40	66·7	1·000						
Caribbean	62	33·3	42	33·3	20	33·3							
Mothers’ immigrant group* (*n* 185)													
Recent immigrants	62	33·5	41	32·8)	21	35·0	0·767						
Settled immigrants	123	66·5	84	67·2	39	65·0							
Mothers’ marital status													
Married/Common-law	124	66·5	79	62·7	45	75·0	0·096	Reference			Reference		
Lone mother (widow, separated, divorced, never married)	62	33·5	47	37·3	15	25·0		0·36	0·15, 0·86	0·021	0·51	0·17, 1·49	0·220
Mothers’ level of education†													
Less than university degree	102	54·8	72	57·1	30	50·0	0·390						
University degree	84	45·2	54	42·9	30	50·0							
Mothers’ employment status													
Currently employed	106	57·0	69	54·8	37	61·7	0·374						
Currently unemployed	80	43·0	57	45·2	23	38·3							
Household income													
Less than 50 000$	104	55·9	74	66·7	30	57·7	0·266						
50 000$ and more	59	31·7	37	33·3	22	42·3							
Missing data‡	23	12·4	–		–								
Social or government assistance (*n* 184)													
Yes	40	21·7	28	22·4	12	20·3	0·752						
No	144	78·3	97	77·6	47	79·7							
Household food insecurity status§ (*n* 181)													
Food secure	102	56·4	70	56·5	32	56·1	0·979						
Food insecure	79	43·6	54	43·5	25	43·9							
Mother’s immigration category (*n* 185)													
Economic immigrant	61	33·3	36	29·3	25	43·9	0·093	Reference			Reference		
Immigrant sponsored by family	50	27·3	34	27·6	16	28·1		0·63	0·25, 1·60	0·331	0·37	0·11, 1·21	0·099
Refugees and asylum seekers	69	37·7	53	43·1	16	28·1		0·71	0·29, 1·72	0·450	0·72	0·23, 2·20	0·558
Born in Canada||	3	1·6	–		–			–		–	–		–

UW, underweight; NW, normal weight; OW, overweight; OB, obesity.

Model 1: child’s gender, mother’s weight status, mother’s marital status, mother’s immigration status.

Model 2: child’s gender, children’s weight status, mother’s weight status, mother’s marital status, mother’s immigration status.

^a,b,c^Represents statistically significant difference between subgroups (*P* < 0·05).

* Mothers’ immigrant group was based on the length of time spent in Canada. In this study, ‘recent immigrants’ refers to immigrants who have spent less than 5 years in Canada and ‘settled immigrants’ refers to immigrants who had spent more than 5 years in Canada.

† Mothers’ education level completed anywhere, whether in Canada or elsewhere.

‡ Missing data were not included in the Pearson *χ*^2^ test.

§ Households were classified as either food secure (score = 0) or food insecure (score = 1–18).

|| Born in Canada not included in the Pearson *χ*^2^ test and the regression models.

¶ Coding for child’s weight perception in the multivariate logistic regression: underweight/normal weight = 0 and overweight/obesity = 1.

** OR were calculated in the logistic regression.

### Factors associated with perceptions of children’s weight status

As shown in Table 1, girls were more likely to be perceived as having overweight than boys (*P* = 0·011). Mothers of children with overweight and obesity were significantly more likely to perceive their children as having overweight than mothers of normal-weight children (*P* < 0·001). Mothers living with obesity, but not with overweight, were similarly more likely to perceive their child as having overweight than normal-weight mothers (*P* = 0·012 and *P* > 0·05, respectively).

The multivariable regression models exploring predictors of maternal perception of children’s weight status are presented in Table 1. A first model was explored in which children’s gender, mothers’ weight status, marital status and immigration category and number of children in the household were included. Model 1 showed that children’s gender, mother’s weight status and marital status were significant predictors of the mothers’ perceptions of children’s weight status, whereas mothers’ immigration status and the number of children in the household were not. This first model accounted for 24 % of the variance. When this model was further adjusted for children’s weight status (model 2), only children’s gender and weight status remained as significant predictors of maternal perception of children’s weight status. Mothers of girls were almost four times more likely to perceive them as having overweight compared with mothers of boys (*P* = 0·041). Additionally, compared with mothers of normal-weight children, mothers of children with overweight (*P* < 0·001) and mothers of children with obesity (*P* < 0·001) were more likely to perceive their children as having overweight. Model 2 accounted for 56 % of the variance.

### Factors associated with concerns about child weight

About half of the participating mothers (48 %) were concerned about their children’s weight status. Children’s measured weight status, mothers’ length of time spent in Canada and household food insecurity status were significantly associated with maternal concerns about child weight (Table 2). Mothers of children with obesity were more likely to be concerned about their children’s weight than mothers of normal-weight children and mothers of children with overweight (*P* < 0·001). Mothers of children with overweight were as likely to be concerned as mothers of normal-weight children. Settled immigrant mothers, mothers who did not have a university degree and mothers living in food-insecure households were more likely to be concerned about their children’s weight status than their counterparts (*P* = 0·046, 0·048 and 0·023, respectively).

**Table 2:** Association between participants’ characteristics and mothers’ concerns about child weight

	Concerns about child weight (*n* 180)										
Participants’ characteristics	Unconcerned *n* 92 (51·1)		Concerned *n* 88 (48·9)			Model 1 Concerns about child weight¶ *n* 157			Model 2 Concerns about child weight¶ *n* 156		
	*n*	Percentage of participants	*n*	Percentage of participants	*P*	OR**	95 % CI	*P*	OR**	95 % CI	*P*
Child’s gender											
Girls	43	46·7	47	53·4	0·371						
Boys	69	53·3	41	46·6							
Number of children in the household											
Two or less	40	43·5	46	52·3	0·238		Reference			Reference	
Three or more	52	56·5	42	47·7		0·58	0·29, 1·19	0·136	0·64	0·21, 1·06	0·069
Child’s age (years)											
Less than 9	41	44·6	37	42·0	0·733						
9 and older	51	55·4	51	58·0							
Mothers’ age (years)											
Less than 40	48	52·2	48	54·5	0·750						
40 and older	44	47·8	40	45·5							
Children’s weight status (*n* 178)											
NW	60	65·6^a^	31	35·6^a^	< 0·001				Reference		
OW	21	23·1^a^	21	24·1^a^					1·96	0·81, 4·72	0·136
OB	10	11·0^b^	35	40·2^b^					6·19	2·31, 16·63	< 0·001
Mothers’ weight status (*n* 166)											
NW	17	19·5	9	11·3	0·099	Reference			Reference		
OW	34	41·5	27	33·8		1·64	0·57, 4·70	0·361	1·47	0·49, 4·44	0·492
OB	32	39·0	44	55·0		2·73	0·99, 7·54	0·052	1·72	0·59, 5·11	0·322
Mothers’ region of origin											
Sub-Saharan Africa	62	67·4	56	63·6	0·596						
Caribbean	30	32·6	32	36·4							
Mothers’ immigrant group*											
Recent immigrants	25	26·9^a^	36	40·9^a^	0·046	1·48	0·67, 3·28	0·333	1·57	0·69, 3·59	0·287
Settled immigrants	68	73·1^b^	52	59·1^b^			Reference			Reference	
Mothers’ marital status											
Married/common law	56	60·9	65	73·9	0·063	Reference			Reference		
Lone mother (widow, separated, divorced, never married)	36	39·1	23	26·1		0·37	0·17, 0·80	0·011	0·47	0·21, 1·06	0·069
Mothers’ education level†											
Less than university degree	44	47·8^a^	55	62·5^a^	0·048	1·79	0·89, 3·61	0·105	1·78	0·83, 3·82	0·137
University degree	48	52·2^b^	33	37·5^b^			Reference			Reference	
Mothers’ employment status											
Currently employed	56	60·9	48	54·5	0·390						
Currently unemployed	36	39·1	40	45·5							
Household income											
Less than 50 000$	50	60·2	51	67·1	0·369						
50 000$ and more	33	39·8	25	32·9							
Missing data‡	–		–								
Social or government assistance											
Yes	17	18·5	20	23·3	0·432						
No	75	81·5	66	76·7							
Household food insecurity status§ (*n* 175)											
Food secure	59	64·1^a^	39	47·0^a^	0·023	Reference			Reference		
Food insecure	33	35·9^b^	44	53·0^b^		2·04	0·97, 4·29	0·060	2·12	0·95, 4·77	0·068
Mother’s immigration category|| (*n* 174)											
Economic immigrant	28	31·8	31	36·0	0·841						
Immigrant sponsored by family	25	28·4	23	26·7							
Refugees and asylum seekers	35	39·8	32	37·2							

UW, underweight; NW, normal weight; OW, overweight; OB, obesity.

Model 1: mother’s weight status, mother’s immigration group, mother’s marital status, mother’s level of education, household food insecurity status.

Model 2: children’s weight status, mother’s weight status, mother’s immigration group, mother’s marital status, mother’s level of education, household food insecurity status.

^a,b,c^Represents statistically significant difference between subgroups (*P* < 0·05).

* Mothers’ immigrant group was based on the length of time spent in Canada. In this study, ‘recent immigrants’ refers to immigrants who have spent less than 5 years in Canada and ‘settled immigrants’ refers to immigrants who had spent more than 5 years in Canada.

† Mothers’ education level completed anywhere, whether in Canada or elsewhere.

‡ Missing data were not included in the Pearson *χ*^2^ test.

§ Households were classified as either food secure (score = 0) or food insecure (score = 1–18).

|| Born in Canada not included in the Pearson *χ*^2^ test and the regression models.

¶ Coding for child’s weight perception in the multivariate logistic regression: unconcerned = 0 and concerned = 1.

** OR were calculated in the logistic regression.

The multivariable regression models exploring predictors of maternal concerns about child weight are presented in Table 2. A first model was explored in which mothers’ weight status, mothers’ marital status, level of education, length of time spent in Canada, number of children in the household and household food insecurity status were included. In this model, which accounted for 17 % of the variance, mothers’ marital status was the only significant predictor of maternal concerns. When this model was further adjusted for children’s weight status (model 2), only children’s weight status significantly predicted mothers’ concerns about child weight. Mothers of children with obesity, but not with overweight, were three times more likely to be concerned about their children’s weight than mothers of normal-weight children (*P* < 0·001 and *P* > 0·05, respectively). Model 2 accounted for 28 % of the variance.

### Children’s diet quality

Children consumed on average 7305 kJ (1746 kcal) during the reference day, with about 37 and 57 % of their total daily energy intake coming from unprocessed or minimally processed foods, and from UPP, respectively (Table 3). There was no difference in children’s energy intake nor the relative consumption of the total daily energy intake provided by unprocessed or minimally processed foods and UPP according to mothers’ concerns about child weight and perceptions of children’s weight status (Table 3).

**Table 3:** Comparison between children’s daily energy intake and intake from NOVA food groups according to maternal concerns and perceptions of children’s weight status

	Daily energy intake (kJ)			Unprocessed or minimally processed foods (%E)			Ultra-processed products (%E)		
	Mean	sd	*P*	Mean	sd	*P*	Mean	sd	*P*
Total	7305	2335		36·53	16·45		56·59	18·66	
Child weight status as perceived by mothers (*n* 186)									
UW/NW	7414	2230	0·365	37·41	16·60	0·288	55·49	18·79	0·246
OW	7079	2540		34·67	16·11		58·89	18·33	
Mothers’ concerns about child weight (*n* 180)									
Concerned	7134	2205	0·292	37·92	16·68	0·287	54·91	19·19	0·225
Unconcerned	7481	2201		35·28	16·49		58·31	18·21	

%E, percentage of energy intake from the NOVA groups; UW, underweight; NW, normal weight; OW, overweight.

## Discussion

To our knowledge, this is the first study to examine predictors of Black immigrant mothers’ perceptions of children’s weight status and concerns about child weight in Canada and to compare children’s diet quality according to these perceptions and concerns about child weight. One-third of participating mothers perceived their children as having overweight, which is higher than what was found in a previous study conducted on parental perceptions of Canadian parents and their children^(58)^. Nearly half of participating mothers were concerned about their children’s weight, which contrasts with other studies reporting that Black parents were less likely to be concerned about child weight than White parents^(28–30)^. Results from the present study also provide evidence that characteristics related to children, mothers and their households influence mothers’ perceptions and concerns about their children’s weight status, as discussed below.

Although there were no significant differences in measured weight status according to children’s gender (data not shown), Black immigrant mothers of girls were more likely to perceive their children as having overweight than mothers of boys. This difference in perception according to children’s gender is consistent with results from one study conducted on a predominantly White population in the USA^(17)^. Ling *et al*. have suggested that gender differences in weight perceptions could be due to body composition as girls tend to have higher amounts of body fat than boys, thus, could be more likely to be perceived as having overweight^(59,60)^. Other authors who have also noted a gender difference in parental perceptions of children’s weight have also suggested that gendered beauty standard and weight stigma applied to girls might also have a role to play in these gendered perceptions^(58,59,61)^. Such gendered perceptions should be further investigated among Black mothers from a variety of cultural backgrounds and their impacts on children’s weight status.

Black immigrant mothers of children with overweight or obesity were significantly more likely to perceive them as having overweight than mothers of normal-weight children. However, the fact that perceptions of overweight and obesity were combined and should be considered along with this interpretation. Indeed, the vast majority of mothers of children with obesity classified their children as having overweight rather than obesity (see online supplementary material, Supplemental Table 4), which could suggest that mothers might perceive excess weight, but only when in high amounts^(6,62)^. Nevertheless, most mothers of children with obesity (78 %) and half of the mothers of children with overweight reported being concerned about their child weight, which is in line with studies reporting that parents are generally concerned about overweight and obesity in children^(19,22)^. Findings also showed that having a child with obesity, but not overweight, was the only significant predictor of maternal concerns about child weight when other factors were taken into account. Altogether, these findings suggest that mothers may express concerns about weight at lower amount of excess weight than the amount required for them to perceive this excess weight or that mothers may avoid labelling children as having obesity given the associated stigma^(63)^.

Black immigrant mothers’ weight status was significantly associated with mothers’ perceptions of children’s weight status, such that mothers with obesity were significantly more likely to perceive their children as having overweight. The literature examining maternal weight status and perceptions of child weight status is equivocal, with some studies reporting that mothers with overweight and obesity were less likely to perceive their children as such^(64,65)^, while others report that mothers with obesity might be more aware of their body size and therefore more sensitive to excess weight of their children^(66,67)^. Mothers’ weight status was a significant predictor of maternal perceptions of children’s weight status only when children’s weight status was not included in the model, which suggests that children’s weight status is a more important determinant of mothers’ perceptions of child weight than mothers’ own weight status. As for concern, mothers’ weight status was not associated with their concerns about child weight, which is in contrast to several studies reporting that parents with overweight or obesity were more likely to be concerned about child weight status than normal-weight parents^(19–21,30,68)^. It is important to note that most of these studies were conducted with White or Latino populations^(19–21,30)^ or had a very small sample of Black/African-American parents^(30,68)^.

In line with previous studies, bivariate analyses also revealed that mothers who lived in food-insecure households were more likely to be concerned about their children’s weight^(69,70)^. Several Canadian studies have shown that children living in food-insecure households, including those from newcomer families, have poorer diet quality^(71,72)^, which can contribute to the development of overweight or obesity^(73)^, thus potentially impacting mother’s concerns about their children becoming overweight in the future. Mothers who were settled immigrants were also more likely to be concerned about their children’s weight. Contrary to recent immigrants, settled immigrants could have had a higher exposition to Western media portraying thin body size/shape^(74)^, and thus, might be more likely to be concerned about child weight. Another possible explanation is that after immigration, some recent immigrants may face economic hardship due to lack of employment or low income^(75)^, which may be of more concern to them than their children’s weight status. Mothers who had less than a university degree were also more concerned about child weight. A study conducted with African Americans showed that parents with lower education levels were less likely to be concerned about their child weight compared to parents with higher education levels^(28)^. Results showed that being a lone mother was also a significant predictor of maternal perceptions of overweight and concerns about child weight when children’s weight status was not taken into account in multivariate models. In Canada, lone mothers are more likely to be unemployed, to have lower education levels and to live in food-insecure households^(76,77)^. Further, as immigrants, participating mothers might face additional challenges such as mental health issues and lower access to healthcare services^(78)^. All these driving factors are related to each other as they are key dimensions of socio-economic position and constitute unfavourable social determinants of health such that mothers experiencing them may not be able to prioritise their child weight status because of competing priorities. Furthermore, these socio-economic characteristics are known to contribute to the development of childhood obesity^(2,79)^, which could explain why these socio-economic factors did not remain significant when implemented along with children’s weight status in multivariate models.

In the present study, children consumed about 57 % of their daily energy intake from UPP, which is similar to the UPP intake of children in Canada (57 %)^(54)^. Such high consumption of UPP is concerning as UPP have poor nutritional value and are not optimal for children’s growth^(54)^. When assessing the relationship between children’s diet quality, as measured with the percentage of energy intake from UPP, and mothers’ perceptions and concerns about child weight, no associations were found. There might be an indirect relationship between these variables throughout feeding practices. Indeed, perceptions and concerns have been reported to influence parental feeding practices, which in turn have an impact on children’s food intake^(3,15,80,81)^. For example, mothers who are concerned about their children’s weight are more likely to use restrictive-feeding practices^(3,15,80,82)^, which have been associated with a higher consumption of restricted energy-dense foods^(80,83)^. Such feeding practices should be avoided given their counterproductive effects on children’s food preferences and intake, and their weight status in the long run^(9,10,81)^. Therefore, studies, both quantitative and qualitative, are needed to better understand the relationships between mothers’ perceptions and concerns, their feeding practices and their children’s diet quality.

### Strengths and limitations

This study has important strengths, in particular the study sample. Indeed, this is the first study to assess perceptions and concerns of child weight among Black immigrant mothers of Sub-Saharan African and Caribbean descent living in Canada, a population that is under-represented in nutrition research. Furthermore, to our knowledge, this study is the first to assess determinants of perceptions of children’s weight status per se (whether accurate or not) along with determinants of misperception of children’s weight status and therefore fills an important knowledge gap. We also assessed children’s diet quality evaluated with the degree of food processing and compared children’s UPP consumption according to Black immigrant mothers’ perceptions and concerns about child weight, which is, to our knowledge, a first in Canada and internationally. Nonetheless, the study limitations should be considered when interpreting the findings. The cross-sectional nature of this study precludes any causality inference. The sample was composed of Sub-Saharan African and Caribbean mothers living in Ottawa, some of which were lone mothers, refugees or asylum seekers, had a low income and were living in food-insecure households. Even though this sample is heterogeneous given the considerable cultural diversity of populations from Sub-Saharan Africa and the Caribbean, Black immigrant mothers from Sub-Saharan Africa and the Caribbean face similar discrimination-related challenges and health inequities in Canada^(33,42,84)^. Due to challenges encountered during the recruitment^(39)^, mothers from these distinct regions were combined into one group for analysis purposes, with an emphasis on immigration status. Similar studies with larger samples of Sub-Saharan African and Caribbean mothers should be conducted to better explore possible cultural differences in the perceptions and concerns of these populations. Therefore, results from this study cannot be generalised to all Black mothers living in Canada. This study was not designed to evaluate immigrant fathers’ perceptions and concerns about child weight even though all parents/guardians’ influences, individually and collectively, contribute to maternal perceptions and concerns about child weight^(85,86)^. The sample was also relatively small, which led to extremely wide CI, especially regarding children’s weight status, and could have led to the overestimation of OR^(87)^. Consequently, OR should be interpreted with caution and larger studies are needed to confirm the present findings. In addition, it is important to note that only one 24-h recall per child was collected, which is not representative of children’s usual diet^(88)^. The NOVA classification system only addresses food processing when evaluating diet quality. Other dimensions of diet quality, such as dietary diversity or meeting the daily nutrient requirements, should also be assessed according to mothers’ perceptions and concerns about child weight to provide a more comprehensive picture. The Figure Rating Scales used to assess mother’s perceptions of their children’s weight status might have not been culturally appropriate for this population as such tools have not yet been developed or validated for children of African descent. Future studies should aim at developing age-appropriate and sex-specific figure rating scales that are culturally appropriate for children of African descent. Studies comparing parental perceptions and concerns of various ethnocultural groups in Canada are also needed, which might help in developing obesity prevention/mitigation intervention programmes that are culturally appropriate.

## Conclusion

Although several factors such as mothers’ weight status, marital status, length of time spent in Canada and household food insecurity were associated with Black immigrant mothers’ perceptions and concerns about child weight, only children’s gender and weight status predicted maternal perceptions and concerns when all factors were taken into account simultaneously. Black immigrant mothers’ perceptions and concerns about child weight, along with their immigration experience, their characteristics and those of their children and household, should be considered by nutrition professionals when developing obesity prevention/mitigation programmes as this might help increase the effectiveness of these programmes. Although mothers’ perceptions and concerns about child weight did not directly influence children’s diet quality as measured by the energy intake from UPP, it is worrisome that children from this study were consuming more than half of their daily energy intake from UPP, which can in itself increase their risk of gaining excess weight over time. Obesity prevention/mitigation programmes should focus on the food system and the social determinants of health so healthy eating and physical activity are possible and easily attainable, as these have positive health outcomes in children regardless of weight status. Further research is needed to assess the relationships between perceptions and concerns about child weight with children’s consumption of UPP. Future studies should aim at examining the long-term effect and potential mechanisms by which perceptions and concerns about the weight of Black mothers from a variety of cultural settings might influence children’s weight status, diet quality and health.

## Supplementary material

For supplementary material accompanying this paper visit https://doi.org/10.1017/S1368980021004729. click here to view supplementary material
