# Supplementary material for: Maternal perceptions and concerns about children’s weight status and diet quality: a study among Black immigrant families
Source: Public Health Nutr. 2021 Dec 6;25(8):2232–45. doi: 10.1017/S1368980021004729 (PMC9991773; doi:10.1017/S1368980021004729)
Supplement: Supplementary file 1 [file S1368980021004729sup001.docx]

**SUPPLEMENTAL MATERIAL**

**FACTORS ASSOCIATED WITH BLACK IMMIGRANT MOTHER’S MISPERCEPTION OF THEIR CHILDREN’S WEIGHT STATUS**

The results presented in this supplemental material aimed to (1) identify factors influencing Black immigrant mothers’ misperceptions of their children’s weight status and (2) compare children’s energy intake and diet quality according to mothers’ misperceptions their children’s weight status.

**METHODS**

**Data analysis**

Pearson Chi-Square tests coupled with Bonferroni post-hoc tests were performed to examine factors associated with maternal misperceptions about child weight. Factors examined were children’s age, gender and weight status, mothers’ age, marital status, weight status, employment status, level of education, immigration admission category, length of time spent in Canada, region of origin, and household income, receipt of social or government assistance, number of children in the household and food insecurity status. Variables with a p-value lower than 0.25 in Pearson Chi-Square tests were included in multivariate logistic regression models ^(1)^ to identify predictors of maternal misperceptions of children’s weight status (1=inaccurate, 0=accurate). T-tests were conducted to compare children’s daily energy intake and diet quality according to maternal misperceptions of children’s weight status.

**RESULTS**

Children’s weight status was misperceived by 51.6% of mothers, with 47.8% underestimating their children’s weight status (Supplemental Table 1). As shown in Supplemental Table 2, only children’s weight status was a significant predictor of mother’s misperceptions of children’s weight status. Mothers of children with overweight and mothers of children with obesity were significantly more like to misperceive (underestimate) their child’s weight status compared to mothers of normal-weight children (both p<0.001). There was no difference in children’s energy intake, nor the relative consumption of the total daily energy intake provided by unprocessed or minimally processed foods and UPP according to mothers’ accurate or inaccurate perceptions of children’s weight status (Supplemental Table 3).

**DISCUSSION**

Almost half of Black immigrants mothers underestimated their children’s weight status, which is consistent with a study conducted in England that included a sample of Black parents ^(2)^. When examining factors associated with mothers’ misperception of children’s weight status, children’s weight status was a significant predictor, which is consistent with the literature ^(7,8)^. Cultural beauty preferences ^(3,4)^, parental lack of knowledge in interpreting what constitutes excess body weight ^(5)^, and acceptance of overweight as the norm by many parents ^(3)^ could explain parental underestimation of overweight or obesity in their children.

**REFERENCES**

1. Bursac Z, Gauss CH, Williams DK, et al. (2008) Purposeful selection of variables in logistic regression. *Source Code Biol. Med.* **3**, 17.

2. Black JA, Park M, Gregson J, et al. (2015) Child obesity cut-offs as derived from parental perceptions: Cross-sectional questionnaire. *Br. J. Gen. Pract.* **65**, e234–e239.

3. Mareno N (2014) Parental perception of child weight: a concept analysis. *J. Adv. Nurs.* **70**, 34–45.

4. Toselli S, Rinaldo N & Gualdi-Russo E (2016) Body image perception of African immigrants in Europe. *Glob. Health* **12**.

5. Dawson AM, Taylor RW, Williams SM, et al. (2014) Do parents recall and understand children’s weight status information after BMI screening? A randomised controlled trial. *BMJ Open* **4**, e004481. British Medical Journal Publishing Group.

6. Seburg EM, Kunin-Batson A, Senso MM, et al. (2014) Concern about Child Weight among Parents of Children At-Risk for Obesity. *Health Behav. Policy Rev.* **1**, 197–208.

7. Blanchet R, Kengneson C-C, Bodnaruc AM, et al. (2019) Factors Influencing Parents’ and Children’s Misperception of Children’s Weight Status: a Systematic Review of Current Research. *Curr. Obes. Rep.* **8**, 373–412.

8. Lundahl A, Kidwell KM & Nelson TD (2014) Parental underestimates of child weight: A meta-analysis. *Pediatrics* **133**, e689–e703.

**Supplemental Table 1.** Accuracy of body shape perception accuracy

|  | **Frequencies (%)**  **n=184** |
| --- | --- |
| **Overestimation** | 6 (3.3) |
| **Adequate perception** | 89 (48.4) |
| **Underestimation** | 89 (48.4) |

**Supplemental Table 2.** Factors associated with body shape perception accuracy

| ***Participants characteristics*** | | ***Misperception (n=184)***  ***(% of participants)*** | | | **Misperception ^6^**  OR^7^[95% CI]  n=160 | **p-value** |
| --- | --- | --- | --- | --- | --- | --- |
|  |  | **Accurate**  **n = 89**  **(48.4)** | **Innaccurate**  **n = 95**  **(51.6)** | ***p-value*** |  |  |
| Child’s gender | | | | |  |  |
|  | *Girls* | 44 (49.4) | 49 (51.6) | 0.772 |  |  |
|  | *Boys* | 45 (50.6) | 46 (48.4) |  |  |  |
| Number of children in the household | | | | | | |
|  | *2 or less* | 42 (47.2) | 44 (46.3) | 0.905 |  |  |
|  | *3 or more* | 47 (52.8) | 51 (53.7) |  |  |  |
| Child’s age (years) | | | | |  |  |
|  | *Less than 9* | 39 (43.8) | 44 (46.3) | 0.734 |  |  |
|  | *9 and older* | 50 (56.2) | 51 (53.7) |  |  |  |
| Mothers’ age (years) | | | | |  |  |
|  | *Less than 40* | 43 (48.3) | 56 (58.9) | 0.148 | Reference |  |
|  | *40 and older* | 46 (51.7) | 39 (41.1) |  | 0.73 [0.33, 1.62] | 0.435 |
| Children’s weight status (n=185) | | | | |  |  |
|  | *NW* | 70 (78.7)^a^ | 24 (25.3)^a^ | <0.001 | Reference |  |
|  | *OW* | 16 (18.0)^b^ | 28 (29.5)^b^ |  | **4.51 [1.84, 11.10]** | **<0.001** |
|  | *OB* | 3 (3.4)^c^ | 43 (45.3)^c^ |  | **43.60 [11.25, 169.00]** | **<0.001** |
| Mothers’ weight status (n=166) | | | | |  |  |
|  | *NW* | 16 (19.3) | 12 (14.5) | 0.065 | reference |  |
|  | *OW* | 36 (43.4) | 25 (30.1) |  | 0.60 [0.20, 1.80] | 0.600 |
|  | *OB* | 31 (37.3) | 46 (55.4) |  | 0.97 [0.34, 2.77] | 0.947 |
| Mothers’ region of origin | | | | |  |  |
|  | *Africa* | 62 (69.7) | 60 (63.2) | 0.351 |  |  |
|  | *Caribbean* | 27 (30.3) | 35 (36.8) |  |  |  |
| Mothers’ immigrant group^1^ (n=185) | | | | |  |  |
|  | *Recent immigrants* | 33 (37.1) | 28 (29.8) | 0.296 |  |  |
|  | *Settled immigrants* | 56 (62.9) | 66 (70.2) |  |  |  |
| Mothers’ marital status | | | | |  |  |
|  | *Married/ Common-law* | 58 (65.2) | 65 (68.4) | 0.640 |  |  |
|  | *Lone mother (widow, separated, divorced, never married)* | 31 (34.8) | 30 (31.6) |  |  |  |
| Mothers’ level of education ^2^ | | | | |  |  |
|  | *Less than university degree* | 47 (52.8) | 54 (56.8) | 0.583 |  |  |
|  | *University degree* | 42 (47.2) | 41 (43.2) |  |  |  |
| Mothers’ employment status | | | | | Reference | 0.528 |
|  | *Currently employed* | 55 (61.8) | 50 (52.6) | 0.209 | 1.30 [0.57, 2.96] |  |
|  | *Currently unemployed* | 34 (38.2) | 45 (47.4) |  |  |  |
| Household income ^3^(n=161) | | | | |  |  |
|  | *less than 50 000$* | 52 (68.4) | 51 (60.0) | 0.267 |  |  |
|  | *50 000$ and more* | 24 (31.6) | 34 (40.0) |  |  |  |
| Social or government assistance | | | | | | |
|  | *Yes* | 18 (20.5) | 21 (22.3) | 0.757 |  |  |
|  | *No* | 70 (79.5) | 73 (77.7) |  |  |  |
| Household food security status ^4^ (n=180) | | | | |  |  |
|  | *Food secure* | 53 (60.2) | 48 (52.7) | 0.313 |  |  |
|  | *Food insecure* | 35 (39.8) | 43 (47.3) |  |  |  |
| Mother’s immigration category^5^ (n=180) | | | | | | |
|  | *Economic* | 33 (38.4) | 28 (30.4) | 0.109 | Reference |  |
|  | *Family class* | 17 (19.8) | 31 (33.7) |  | 2.74 [0.96, 7.85] | 0.061 |
|  | *Refugees* | 36 (41.9) | 33 (35.9) |  | 1.97 [0.74, 5.26] | 0.176 |

OR, odd ratios CI, confidence interval UW, underweight NW, normal weight OW, overweight OB, obese

^1^ Mothers’ immigrant group was based on length of time spent in Canada. In this study, “recent immigrants” refers to immigrants who has spent less than 5 years in Canada and “settled immigrants” refers to immigrants who had spent more than 5 years in Canada.

^2^ Mothers’ level of education completed anywhere, whether in Canada or elsewhere.

^3^ Missing data were not included in the Pearson chi-square test.

^4^ Households were classified as either food secure (score=0) or food insecure (score=1-18).

^5^ Born in Canada not included in the Pearson chi-square test and the regression models.

^a,b,c^ represents statistically significant difference between subgroups (p<0.05).

^6^ Coding for child’s weight perception in the multivariate logistic regression: Accurate=0 and Inaccurate=1.

^7^ Odds Ratio were calculated in the logistic regression.

Model: Children’s weight status, Mothers’age, weight status, employment status and immigration category

**Supplemental Table 3.** Comparison between children’s daily energy intake and intake from NOVA food groups according to mothers’ body shape perception accuracy

|  |  | **Daily energy intake (kcal)**  **(M ± SD)** | **p-value** | **Unprocessed or minimally processed foods (%E)**  **(M ± SD)** | **p-value** | **Ultra-processed products (%E)**  **(M ± SD)** | **p-value** |
| --- | --- | --- | --- | --- | --- | --- | --- |
| **Body shape perception accuracy (n=184)** | | | | | | | |
|  | Accurate | 1780 ± 553 | 0.466 | 38.86 ± 17.20 | 0.077 | 54.33 ± 18.84 | 0.140 |
|  | Inaccurate | 1720 ± 566 |  | 34.56 ± 15.61 |  | 58.41 ± 18.69 |  |

M mean, SD standard deviation, %E percentage of energy intake from the NOVA groups, UW, underweight NW, normal weight OW, overweight OB, obese

**Supplemental Table 4.** Children’s measured weight status and perception of children’s weight status (n=185)

|  | **Perception of children’s weight status** | | | | **Total** |
| --- | --- | --- | --- | --- | --- |
| **Children’s measured weight Status** | **Underweight** | **Normal weight** | **Overweight** | **Obesity** |  |
| **Normal Weight** | 18 | 70 | 6 | 0 | 95 |
| **Overweight** | 2 | 26 | 16 | 0 | 44 |
| **Obesity** | 0 | 9 | 34 | 3 | 46 |
| **Total** | 20 | 105 | 56 | 3 | 184 |
